# Symptom severity at first digital self-assessment is not associated with greater subsequent booking of professional care on a Saudi mental health platform: a cohort study of 5,524 users

**DOI:** 10.3389/fpsyt.2026.1928345

**Published:** 2026-10-27

**Authors:** Abdulmajeed A. Alkhamees

**Affiliations:** Department of Psychiatry, College of Medicine, Qassim University, Buraydah, Saudi Arabia

**Keywords:** cohort study, digital mental health, GAD-7, help-seeking, PHQ-9, Saudi Arabia, symptom severity

## Abstract

**Background:**

Digital mental health platforms routinely deliver self-administered symptom scales on the assumption that users with more severe symptoms are more likely to enter professional care. That assumption is rarely tested with linked participant-level data, and almost never in the Arabic-speaking Gulf.

**Objective:**

To examine whether symptom severity at a user’s first self-assessment predicts booking a professional session within 90 days, across four symptom domains, on a Saudi commercial digital mental health platform.

**Methods:**

Retrospective cohort analysis of 10,100 first assessments — depression, generalized anxiety, panic, and obsessive-compulsive scales — contributed by 5,524 unique users of the Talbinah platform and linked to demographic and session-booking records through a pseudonymous (SHA-256 hashed) user identifier. The primary outcome was any session booked within 90 days after the assessment. Severity was banded by the established cut-offs for the PHQ-9 and GAD-7, and by percentage of scale maximum for the panic and obsessive-compulsive measures, for which no severity thresholds are established. Per-scale multivariable logistic regression estimated adjusted odds ratios (aOR) for severity band (reference None/Minimal), adjusting for age, sex, and device type. Pre-specified sensitivity analyses used 30- and 180-day windows, an attended-session outcome, percentage-of-maximum banding applied to all four scales, and a subset with no prior booking (incident help-seeking). Reporting followed STROBE.

**Results:**

Users were young (mean age 26.7 years, SD 8.1) and predominantly female (78.1%); 16.6% of first assessments were followed by a booking within 90 days. No severity band on any of the four scales was associated with higher odds of booking (severe vs None/Minimal: depression aOR 0.71, 95% CI 0.51–1.01; generalized anxiety 0.76, 0.49–1.16; panic 0.83, 0.52–1.31; obsessive-compulsive 1.01, 0.59–1.74). One severity contrast was statistically significant, in the opposite direction: moderate depression was associated with lower odds of booking (0.66, 95% CI 0.45–0.97). Older age (aOR ~1.2 per decade) and iOS device use (1.4–2.0) were associated with booking across scales. This absence of a positive severity–booking association was consistent across 30-, 90-, and 180-day windows, the attended-session outcome, and percentage-of-maximum banding; a nominal association for moderate anxiety seen under percentage banding did not persist with validated GAD-7 cut-offs. Estimates for the panic and obsessive-compulsive scales, which lack established severity thresholds, are exploratory.

**Conclusions:**

On a Saudi digital mental health platform, greater self-reported symptom severity at first assessment was not followed by higher subsequent booking of professional care, consistently across specifications, and for moderate depression was associated with lower odds of booking; demographic and access-related attributes showed stronger associations with booking than symptom load did. Self-screening on its own appears insufficient as a pathway into care.

## 1 Introduction

Mental disorders are common in Saudi Arabia, and most affected people never reach care. The Saudi National Mental Health Survey estimated lifetime prevalence of any DSM-IV disorder at 34.2% and 12-month prevalence at 20.2%, with anxiety disorders the most prevalent class (1, 2). National screening in 2022 identified substantial proportions of adults at risk for depression and generalized anxiety alongside very low treatment rates (3). Against this treatment gap, Arabic-language digital mental health platforms have expanded across the Gulf, offering direct-to-consumer self-assessment and booking with licensed providers (4–7).

Self-administered scales such as the PHQ-9 and GAD-7 are a common first point of contact on these platforms, and it is generally assumed that users with more severe symptoms are more likely to move from self-assessment to professional help-seeking. That assumption underpins the use of self-screening as a triage and engagement tool. It is rarely tested with linked, participant-level platform data, and almost never in the Arabic-speaking Gulf, where stigma, cost, and service supply are prominent correlates of help-seeking (2–4).

Using pseudonymously linked records from a Saudi commercial platform, this cohort study tests whether symptom severity at a user’s first self-assessment predicts booking a session within 90 days, separately for depression, generalized anxiety, panic, and obsessive-compulsive symptoms, adjusting for age, sex, and device, with pre-specified sensitivity analyses including a subset who had not previously booked (incident help-seeking).

## 2 Methods

### 2.1 Setting and platform

Talbinah is a Saudi Arabia-based Arabic-language commercial digital mental health platform that offers a library of self-administered psychometric scales and direct booking of sessions with licensed providers. Each scale is presented in Arabic; users may complete a scale before booking, after a session, or independently of any booking. Each completed attempt is stored with a raw score, a percentage-of-maximum score, a severity-band label, and a timestamp.

### 2.2 Design and data sources

This is a retrospective cohort analysis at the level of the user’s first assessment on a given scale. Three de-identified platform tables were linked: a psychometric table, a user-level demographic table, and a session-booking table. Records were linked by a single SHA-256 pseudonymous user identifier common to all tables; direct identifiers were removed by the platform, but each user’s records remain internally linkable. The extract spanned September 2023 to April 2026. No re-identifiable information was accessed.

### 2.3 Participants

Users were included if they completed at least one of the four analyzed scales and had valid demographic data (internal/staff accounts excluded; recorded age 18–100 years, consistent with the platform’s Terms of Service, which require users to be 18 years or older at registration; recorded sex of Female or Male, the only two values present). The unit of analysis was the user-by-scale first assessment (the earliest completed attempt per user per scale). The observed age range was 18–80 years. Registration under the platform’s Terms of Service is the basis of consent for all users (see Ethics, Section 2.8).

### 2.4 Measures and severity banding

Four instruments were analyzed: the PHQ-9 for depression (9 items, 0–27) (8); the GAD-7 for generalized anxiety (7 items, 0–21) (9); the panic module of the Patient Health Questionnaire, the Arabic version of which has documented reliability and validity in a Saudi sample (10); and an obsessive-compulsive scale derived from the Y-BOCS (11). For the PHQ-9 and GAD-7, severity followed the instruments’ established cut-offs (None/Minimal 0–4, Mild 5–9, Moderate 10–14, Severe ≥15). For the panic and obsessive-compulsive measures, no severity thresholds are established for the versions implemented on this platform, so severity was banded by percentage of scale maximum (None/Minimal <25%, Mild 25 to <50%, Moderate 50 to <75%, Severe ≥75%); percentage-of-maximum banding was additionally applied to all four scales as a sensitivity analysis. The percentage-of-maximum transformation follows the percentage-of-maximum-possible (POMP) scoring convention, which places scores from different instruments on a common 0–100 metric for comparison (12); the quartile cut-points used here (<25%, 25 to <50%, 50 to <75%, ≥75%) are an equal-width convention chosen for this reason and are not themselves clinically validated thresholds. Two distinctions matter for interpretation. Documented reliability and validity for the Arabic panic module (10) is not the same as an established severity threshold, and the obsessive-compulsive scale is a Y-BOCS-derived instrument that has not been formally validated in this implementation. Findings for these two scales are therefore treated as exploratory throughout, with primary inference resting on the PHQ-9 and GAD-7 results.

### 2.5 Outcome

The primary outcome was any session booked within 90 days after the first-assessment date, defined from the booking-creation timestamp. Booking, rather than attendance, was chosen as the primary outcome because it is the first actionable step directly following the assessment and is recorded for every user regardless of subsequent scheduling or cancellation, whereas attendance conflates the severity–booking association of interest with additional, unmeasured determinants of keeping a scheduled session. Pre-specified sensitivity outcomes were bookings within 30 and 180 days, and an attended session (session started) within 90 days; the attended-session analysis, reported in Section 3.3, addresses attendance directly as a more clinically meaningful indicator and produced results consistent with the primary outcome.

### 2.6 Covariates

Models adjusted for age (per 10-year increment), sex (female vs male), and device type (iOS vs other), the participant-level attributes available in the linked extract.

### 2.7 Statistical analysis

For each scale, a multivariable logistic regression estimated adjusted odds ratios (aOR) with 95% confidence intervals (CI) for severity band (reference None/Minimal) and for the covariates. Each user contributed at most one first assessment per scale, so observations within a model are independent; because the same users may appear in more than one scale-specific model, the four models are not independent of one another. Event counts ranged from 233 to 664 per scale, giving moderate precision: for the highest severity band, the upper confidence limits ranged from approximately 1.0 (depression) to 1.7 (obsessive-compulsive), so the data are most consistent with the absence of a moderate-to-large positive severity–booking association, while smaller positive associations cannot be excluded, particularly for the panic and obsessive-compulsive scales, whose intervals were the widest. Each model estimated three severity contrasts against the reference band across four scales, and no formal multiplicity correction was applied, so an isolated interval excluding 1 is interpreted with corresponding caution. Sensitivity analyses repeated each model across the alternative outcome windows, the attended-session outcome, percentage-of-maximum banding, and the incident-help-seeking subset. Analyses used Python 3 with the statsmodels library. Reporting followed STROBE for cohort studies (13).

### 2.8 Ethics

The study was approved by the Talbinah Platform Ethical Review Board (reference T-D-1910, 18 March 2026; Chairman Dr. Mohammed Al-Ghusn, an ethics committee constituted by the platform to review secondary uses of its data and operating independently of this analysis. Users agree at registration to the platform’s Terms of Service, which permit the secondary use of de-identified, routinely collected records for service improvement and research; this registration-based consent, which applies to every registered user, covered the present secondary analysis. Because the work was a secondary analysis of fully de-identified records from which direct identifiers had been removed by the platform before access, separate university institutional review board approval was not required under applicable local regulations, and no identifiable user data were accessed. Review by the platform’s own committee, rather than an external academic institutional review board, is acknowledged as a limitation (Section 4.5).

## 3 Results

### 3.1 Cohort characteristics

The analytic cohort comprised 5,524 unique users contributing 10,100 first assessments across the four scales. Users were young (mean age 26.7 years, SD 8.1; range 18–80), predominantly female (78.1%), and mostly using iOS devices (80.9%). Overall, 16.6% of first assessments were followed by a booking within 90 days (13.8% within 30 days, 18.0% within 180 days; 13.9% attended a session within 90 days). Severity distributions and booking rates by scale are shown in **Table 1**.

**Table 1:** Cohort characteristics and outcomes by scale (10,100 first assessments from 5,524 users; overall mean age 26.7 years [SD 8.1], 78.1% female, 80.9% iOS).

Characteristic	Depression	GAD	Panic	OCD
First assessments, n	4,607	2,638	1,662	1,193
None/Minimal, n (%)	239 (5.2)	150 (5.7)	152 (9.1)	340 (28.5)
Mild, n (%)	596 (12.9)	451 (17.1)	356 (21.4)	381 (31.9)
Moderate, n (%)	923 (20.0)	781 (29.6)	670 (40.3)	357 (29.9)
Severe, n (%)	2,849 (61.8)	1,256 (47.6)	484 (29.1)	115 (9.6)
Booked within 90 d, %	14.4	17.9	18.4	19.5
Attended within 90 d, %	11.8	15.5	15.6	16.0

Depression and GAD banded by validated cut-offs; panic and OCD by percentage of maximum.

GAD, generalized anxiety; OCD, obsessive-compulsive.

### 3.2 Symptom severity and subsequent booking

After adjustment for age, sex, and device type, no severity band on any of the four scales was associated with higher odds of booking within 90 days (**Table 2**). No severity estimate lay above 1 with a confidence interval that excluded 1. One severity contrast was statistically significant, and it ran opposite to the hypothesized direction: users in the moderate depression band had lower odds of booking than users in the None/Minimal band (aOR 0.66, 95% CI 0.45–0.97). The severe depression estimate pointed the same way with an interval that included 1 (0.71, 0.51–1.01). For depression, then, the pattern is flat to inverse rather than graded upward. Given the number of severity contrasts estimated and the absence of a monotonic dose–response gradient (Section 2.7), this single association is reported as observed and interpreted cautiously. Across scales, the attributes consistently associated with booking were older age (aOR approximately 1.2 per 10-year increment) and use of an iOS device (aOR 1.4–2.0); sex was not associated with booking. **Figure 1** displays the severity odds ratios across scales.

**Table 2:** Adjusted odds ratios (95% CI) for booking within 90 days, per scale (reference for severity = None/Minimal).

Predictor	Depression	GAD	Panic	OCD
Mild	0.88 (0.59–1.30)	0.91 (0.57–1.45)	1.03 (0.64–1.64)	1.02 (0.70–1.48)
Moderate	0.66 (0.45–0.97)	0.97 (0.62–1.50)	0.79 (0.51–1.23)	1.04 (0.71–1.53)
Severe	0.71 (0.51–1.01)	0.76 (0.49–1.16)	0.83 (0.52–1.31)	1.01 (0.59–1.74)
Age (per 10 y)	1.19 (1.08–1.32)	1.24 (1.10–1.40)	1.12 (0.96–1.32)	1.21 (1.01–1.44)
Female (vs male)	1.01 (0.82–1.23)	0.93 (0.73–1.19)	0.82 (0.60–1.12)	0.93 (0.66–1.31)
iOS (vs other)	1.42 (1.13–1.80)	1.96 (1.46–2.62)	1.82 (1.26–2.62)	1.45 (0.99–2.11)

Among the severity contrasts, only moderate depression has an interval excluding 1 (0.66, 95% CI 0.45–0.97); the remaining intervals excluding 1 are in the age and device rows. Per-scale n = 4,607 (Depression), 2,638 (GAD), 1,662 (Panic), 1,193 (OCD).

**Figure 1:**
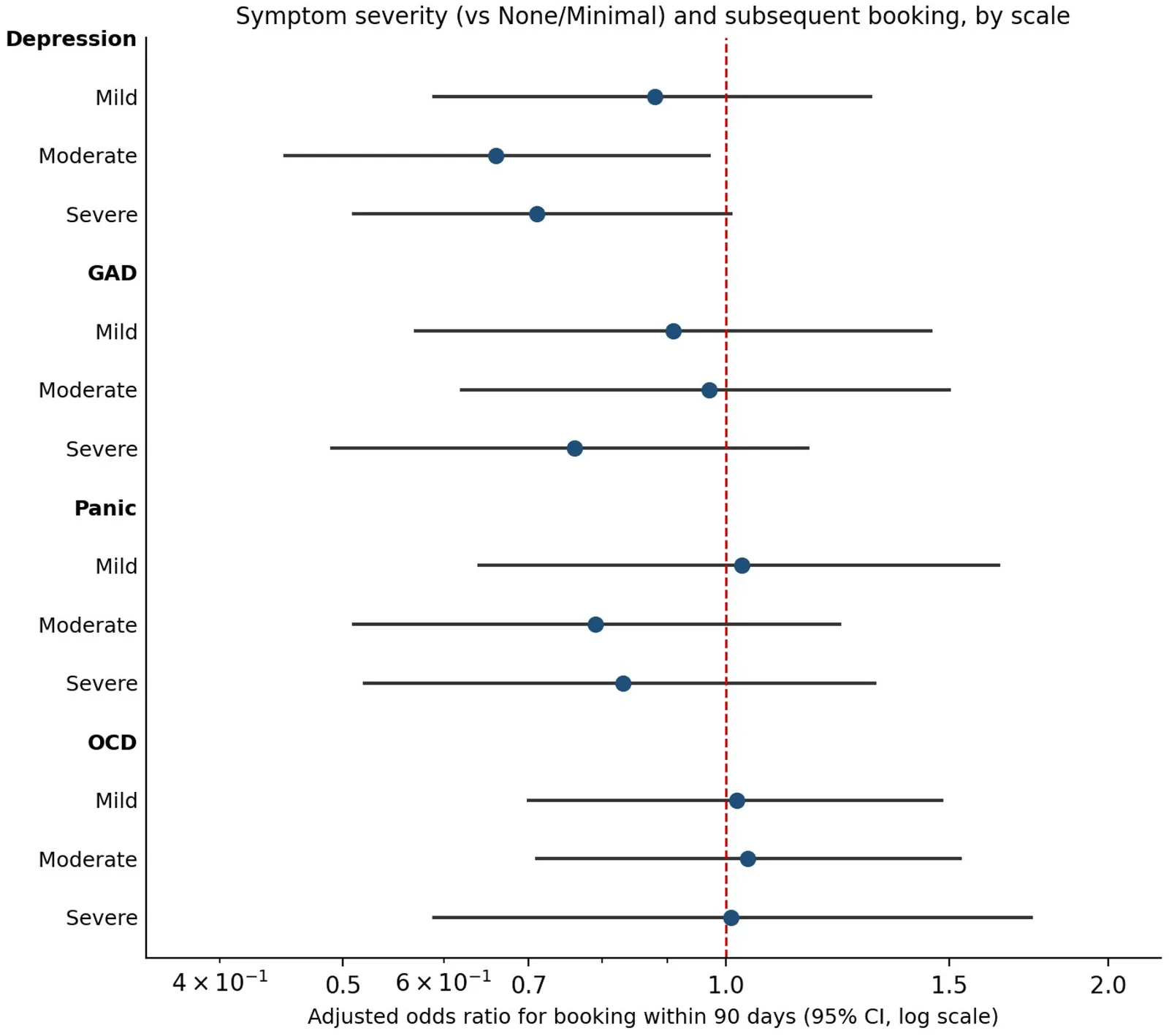
Adjusted odds ratios (95% CI) for booking within 90 days by severity band (reference None/Minimal), per scale. The dashed line marks OR = 1. No severity estimate lies above the line with an interval excluding it; the single interval that excludes 1 — moderate depression, 0.66 (95% CI 0.45–0.97) — lies below it.

### 3.3 Sensitivity analyses

The absence of a positive severity–booking association was stable across specifications. Using a 30- or 180-day window, and using an attended-session rather than booked-session outcome, severity estimates remained close to the null on all four scales (for example, depression severe-vs-None aOR 0.76 at 30 days, 0.69 at 180 days, and 0.78 for attended sessions). Applying percentage-of-maximum banding to all four scales produced a nominal association for moderate generalized anxiety in the incident-help-seeking subset (aOR 3.34, 95% CI 1.27–8.75). It did not persist under the validated GAD-7 cut-offs in the same subset (aOR 1.78, 95% CI 0.67–4.78) and is therefore not interpreted as a reliable association. Restricting to users with no booking before the assessment (incident help-seeking; 1,876 users, 3,336 assessments; 10.3% booked within 90 days) did not produce a severity association on any scale.

## 4 Discussion

### 4.1 Principal findings

Among 5,524 users of a Saudi digital mental health platform, greater self-reported symptom severity at first self-assessment did not predict a higher likelihood of booking a professional session within 90 days, and that pattern held across banding schemes, outcome windows, an attended-session outcome, and the incident-help-seeking subset. The attributes reliably associated with booking were older age and iOS device use rather than symptom load. For depression, higher severity was associated with lower odds of booking, statistically significantly so in the moderate band. Findings for the panic and obsessive-compulsive scales are exploratory, given the absence of established severity thresholds for those measures.

### 4.2 Interpretation

Three explanations fit this pattern, and they carry different implications. The first is range restriction: the platform population is self-selected and already symptomatic, so the severity distribution may be compressed and offer little gradient against which booking can vary; if so, a positive severity–booking association could still be present in a general-population sample screened under the same protocol, and the present result would be specific to populations conditioned on platform entry. The second is that booking tracks resources and access rather than symptom load: age and device type were the two attributes associated with booking, and both plausibly index means and autonomy — age the capacity to decide on and pay for care, device type socioeconomic position or depth of platform use — while neither indexes severity. The third is that users with the highest scores are the least likely to take this particular route: they may disengage, seek in-person or urgent care outside the platform, or use repeated self-assessment as self-monitoring rather than as a precursor to booking; the inverse association for moderate depression is compatible with this account, although one contrast cannot establish it. The available covariates cannot arbitrate among these explanations, which would require measures the extract does not contain — symptom-specific functional impairment, ability to pay, and care received off-platform — and, for the first, a sample not conditioned on platform entry.

### 4.3 Comparison with prior work

The finding is consistent with a wider literature in which clinical need alone is a weak predictor of mental health service use, and with regional evidence that stigma and access barriers are associated with help-seeking over and above symptom severity (2–4, 6). It also cautions against treating digital self-screening as a self-sufficient pathway into care: on this platform, high self-screened severity was not followed by higher booking.

### 4.4 Implications

If symptom severity does not distinguish the users who go on to book from those who do not, passive screening is unlikely to close the treatment gap on its own. Platforms and services may need active linkage — proactive outreach, reduced financial and procedural friction, and targeted follow-up of high-severity self-screeners — to connect measured distress to care.

### 4.5 Limitations

This observational analysis cannot establish causation, and residual confounding is likely: symptom-specific functional impairment, treatment history beyond prior booking on the platform, financial means, and social support were unavailable, and device type is only a crude proxy for socioeconomic position. Users already receiving mental health care outside the platform are not identifiable in these data; if such off-platform care is more common among more symptomatic users, and reduces their need to book on-platform, this unmeasured confounding would bias the severity–booking association toward the null or negative direction, offering one plausible contributor to the inverse association observed for moderate depression. Conversely, if the most functionally impaired users face the greatest practical barriers to booking regardless of platform care-seeking elsewhere, the same negative bias would follow; both mechanisms would understate rather than overstate any true positive severity–booking association. Severity relied on self-report, and the panic and obsessive-compulsive measures were adapted or derived and banded by percentage of maximum rather than by established thresholds, so their estimates are exploratory. The data come from a single commercial platform in one country, which limits generalizability. Ethical review was conducted by the platform’s own ethics committee rather than an external academic institutional review board. Precision, finally, was moderate rather than high: the analyses are most consistent with the absence of a moderate-to-large positive severity association but cannot exclude small positive associations, particularly for the panic and obsessive-compulsive scales, whose confidence intervals were the widest.

## 5 Conclusion

On a Saudi digital mental health platform, greater severity of self-reported symptoms at first assessment did not predict a higher likelihood of subsequently booking professional care, consistently across specifications; demographic and access-related attributes showed stronger associations with booking. Moving self-screened distress into care is likely to require active linkage rather than screening alone.

## Data Availability

The datasets presented in this article are not readily available due to platform data-governance restrictions. Requests to access the datasets should be directed to the corresponding author(s).
